# Distinct Phenotypes Induced by Different Degrees of Transverse Aortic Constriction in C57BL/6N Mice

**DOI:** 10.3389/fcvm.2021.641272

**Published:** 2021-04-22

**Authors:** Haiyan Deng, Lei-Lei Ma, Fei-Juan Kong, Zengyong Qiao

**Affiliations:** ^1^Department of Cardiovascular Medicine, Shanghai Jiao Tong University Affiliated Sixth People's Hospital South Campus, Shanghai, China; ^2^Department of Cardiology, Shanghai Institute of Cardiovascular Diseases, Zhongshan Hospital, Fudan University, Shanghai, China; ^3^Department of Endocrinology and Metabolism, Shanghai General Hospital, Shanghai Jiao Tong University School of Medicine, Shanghai, China; ^4^Department of Endocrinology and Metabolism, Xuhui District Central Hospital of Shanghai, Shanghai, China

**Keywords:** cardiac hypertrophy, transverse aortic constriction, C57BL/6N mice, cardiac fibrosis, heart failure

## Abstract

The transverse aortic constriction (TAC) model surgery is a widely used disease model to study pressure overload–induced cardiac hypertrophy and heart failure in mice. The severity of adverse cardiac remodeling of the TAC model is largely dependent on the degree of constriction around the aorta, and the phenotypes of TAC are also different in different mouse strains. Few studies focus on directly comparing phenotypes of the TAC model with different degrees of constriction around the aorta, and no study compares the difference in C57BL/6N mice. In the present study, C57BL/6N mice aged 10 weeks were subjected to sham, 25G TAC, 26G TAC, and 27G TAC surgery for 4 weeks. We then analyzed the different phenotypes induced by 25G TAC, 26G TAC, and 27G TAC in c57BL/6N mice in terms of pressure gradient, cardiac hypertrophy, cardiac function, heart failure situation, survival condition, and cardiac fibrosis. All C57BL/6N mice subjected to TAC surgery developed significantly hypertrophy. Mice subjected to 27G TAC had severe cardiac dysfunction, severe cardiac fibrosis, and exhibited characteristics of heart failure at 4 weeks post-TAC. Compared with 27G TAC mice, 26G TAC mice showed a much milder response in cardiac dysfunction and cardiac fibrosis compared to 27G TAC, and a very small fraction of the 26G TAC group exhibited characteristics of heart failure. There was no obvious cardiac dysfunction, cardiac fibrosis, and characteristics of heart failure observed in 25G TAC mice. Based on our results, we conclude that the 25G TAC, 26G TAC, and 27G TAC induced distinct phenotypes in C57BL/6N mice.

## Introduction

Heart failure (HF) is still one of the leading causes of mortality worldwide, and the prevalence of HF continues to rise over time. Just between the years 2013 and 2016, there were about 6.2 million adults diagnosed with HF in America (1), which is a great loss to people's health and the economy. As a result, it is more important to better understand the development of HF and find new therapeutic targets. The development of HF is characterized by a process of adverse cardiac remodeling (2), and there are three major patterns of cardiac remodeling: pressure overload–induced concentric hypertrophy, volume overload–induced eccentric hypertrophy, and mixed load–induced post-myocardial-infarct remodeling (3). Heart disease animal models play an important role in studying these remodeling processes as well as in preclinical studies. The validity and accuracy of animal models are necessary for the mechanism study of HF and for new drug development as well (4, 5).

Hypertension is one of the most important risk factors in the development of HF. To learn the mechanisms of pressure overload–induced cardiac hypertrophy, the transverse aortic constriction (TAC) model was first built by Rockman et al. (6). Over decades of development, several improved TAC models with minimal invasiveness and low mortality have been developed by other research groups (7–9), and this made the TAC model more effective and accurate. Animals subjected to TAC go through cardiac hypertrophy, cardiac fibrosis, a limited amount of inflammation, and eventually develop to cardiac dilation and HF. The severity of adverse cardiac remodeling induced by TAC largely relies on the degree of constriction of the aorta and the duration of aortic constriction correlated to the degree of adverse remodeling (10). The most common needle size for constriction in TAC is 27 gauge (27 G, outer diameter 0.41 mm) (4, 5, 10) although there are limited studies using 25-gauge (25 G, outer diameter 0.51 mm) (11) and 26-gauge (26 G, outer diameter 0.46 mm) needles (12). Compared with bigger size needles, smaller needles can create a narrower constriction loop, which induces severe adverse remodeling and higher mortality (13). Besides the severity of constriction, the species of animals and genetic background are also responsible for the variability of TAC response. Therefore, a better understanding of the accurate response of specific animal strains and specific constriction to TAC is very important for the research on HF. However, very few studies focus on the direct comparison of phenotypes of the TAC model induced by different needle sizes in great detail (13). Also, the phenotype comparison of TAC response induced by different needle sizes in C57BL/6N mice has not been reported to date. The potentially different adverse cardiac remodeling induced by varying degrees of pressure overload in C57BL/6N mice remains unknown.

In the present study, we compare the different response to TAC induced by 25 G, 26 G, and 27 G needles in C57BL/6N mice, and we analyze cardiac hypertrophy, cardiac function, and cardiac fibrosis changes in response to varying constriction degrees. Our study may provide deep insight for a pressure overload–induced heart failure study in C57BL/N mice.

## Methods

### Animals

The male C57BL/6N mice used in this study were obtained from Shanghai Laboratory Animal Center (Chinese Academy of Sciences, Shanghai, China). All the animal experiments were conducted in compliance with the Guide for the Care and Use of Laboratory Animals published by the U.S. National Institutes of Health (the 8th edition, NRC 2011). All the *in vivo* experiments were approved by the Animal Care and Use Committee of Fudan University.

### TAC Surgery

Thirty male C57BL/6N mice aged 10 weeks were randomly divided into four groups: Sham (*n* = 6), 25 G TAC (*n* = 8), 26 G TAC (*n* = 8), and 27 G TAC (*n* = 8). All TAC group mice were subjected to minimally invasive TAC surgery as described elsewhere (14). Mice were anesthetized with 1% pentobarbital sodium (50 mg/kg) through intraperitoneal injection. The hair on the neck and chest were removed using a depilatory agent, and then the surgery area was disinfected with betadine and alcohol. A 0.5–1.0 cm longitudinal skin incision was made at the level of the suprasternal notch, and then a 2–3 mm longitudinal sternum cut was made to locate the thymus and aorta. The aorta between the origin of the right innominate and left common carotid arteries was tied with a bent 25 G, 26 G, or 27 G needle with a 6-0 silk suture, and then the needle was quickly removed after ligation. The sham mouse surgery was under the same procedures except that the artery was not ligated. After surgery, the mice were allowed to fully recover on a warming pad and housed in standard housing condition.

### Echocardiography

Echocardiography analysis was performed using the Vevo 2100 imaging system (VisualSonics, Canada) as described in a previous study (15). Briefly, the mice were anesthetized with isoflurane (0.5–4%), and parasternal long- and short-axis views in B- and M-Mode were recorded when the heart rate of the mice was maintained at 450–550 bpm. To access the peak pressure gradient induced by TAC, pulsed-wave Doppler was applied to the aortic arch as described elsewhere (16). The pulsed-wave Doppler was used to measure blood velocities in either TAC or sham mice. The peak pressure gradient was calculated with the pulsed-wave peaks by using the modified Bernoulli equation (Pressure gradient = 4^*^velocity^2^) (16). All measurements were averaged with three measurements per variable animal.

### Tissue Collection

Mice were anesthetized with 5% isoflurane. After being fully anesthetized, the heart was quickly excised and washed with ice-cold PBS. After being weighed, the heart was cut into three parts. The middle part was put into 4% polyformaldehyde solution for histological analysis, and the top and bottom parts were quickly put into liquid nitrogen and transferred to a −80° freezer later. The lungs and tibia length were also weighed and measured.

### Histological Staining

Cardiomyocyte size and extent of LV fibrosis were measured using hematoxylin and eosin (H&E) and Masson's trichrome staining, respectively. After being fixed with 4% polyformaldehyde, the heart was finally embedded into paraffin. The heart-embedded paraffin blocks were cut into 5-μm sections. Then, the sections were stained with H&E and Masson's trichrome. At least five sections of each heart were examined, and at least five random images were captured at each section. The images were analyzed by using the Image-Pro Plus 5.0 image analysis system (Media Cybernetics, Rockville, MD).

### Real-Time Polymerase Chain Reaction (RT-PCR)

The gene expression level of col-1, col-3, and TGF-β were measured using RT-PCR. Total RNA was extracted from frozen heart tissue by using the TRIzol reagent (Invitrogen, Carlsbad, CA), and 1 μg total RNA was used for reverse transcription to synthesize cDNA by using TOYOBO RT-PCR kit (TOYOBO, Japan). SYBR Premix Ex Taq kit (Cat#: RR420A, TaKaRa, Japan) was used in the RT-PCR reaction for relative quantification of RNA. The primers we used were synthesized by Sangon Biotech (Shanghai, China), and the sequences of the primers are as follows: col-1 (Forward: CAACCTCAAGAAGTCCCTGC, Reverse: AGGTGAATCGACTGTTGCCT), col-3 (Forward: CACCCCTCTCTTATTTTGGCAC, Reverse: AGACTCATAGGACTGACCAAGGTAGTT), TGF-β (Forward: GGCGGTGCTCGCTTTGTA, Reverse: GCGGGTGACTTCTTTGGC), ANP (Forward: CTGCTTCGGGGGTAGGATTG, Reverse: GCTCAAGCAGAATCGACTGC), BNP (Forward: GAGGTCACTCCATCCTCTGG, Reverse: GCCATTTCCTCCGACTTTTCTC), GAPDH (Forward: AACAAGCAACTGTCCCTGAGC, Reverse: GTAGACAGAAGGTGGCACAGA). GAPDH was used as a reference gene.

### Western Blot

Total proteins were extracted from heart tissues and quantified by using the bicinchoninic acid protein assay. According to the molecular weight of the target proteins, the protein samples were separated in 10% SDS-PAGE and then transferred to Immobilon-P polyvinylidene fluoride (PVDF) membranes. After being blocked with Western blocking buffer, the membranes were incubated overnight at 4°C with primary antibodies: TGF-β (1:1,000), COL1A1 (1:1,000), phosphorylated ERK (1:5,000), and ERK (1:5,000) (Cell Signaling Technology, Danvers, MA, USA) and then incubated with horseradish peroxidase (HRP)-conjugated secondary antibodies (1:1,000) for 1 h at room temperature. After interaction with a Pro-Light chemiluminescent detection kit (Tiangen Biotech Inc., Beijing, China), the proteins of the membranes were detected using the LAS-3000 imaging system (FUJIFILM Inc., Tokyo, Japan), and the results were analyzed with ImageJ software (National Institutes of Health, Bethesda, MD, USA).

### Statistical Analysis

All the data in the present study are expressed as mean ± standard error. All the data statistics analyses were performed by GraphPad Prism 8.0 (GraphPad Prism Software, CA, USA). For comparison of more than two groups, one-way ANOVA followed by Tukey's post-test was used. For two-factor design experiments, two-way ANOVA followed by Tukey's multiple comparisons test was performed. Statistical significance was presented by repeat symbols: a single symbol means *p* ≤ 0.05; double symbols mean *p* ≤ 0.01; triple symbols mean *p* ≤ 0.001.

## Results

### Pressure Gradient

**Figure 1:**
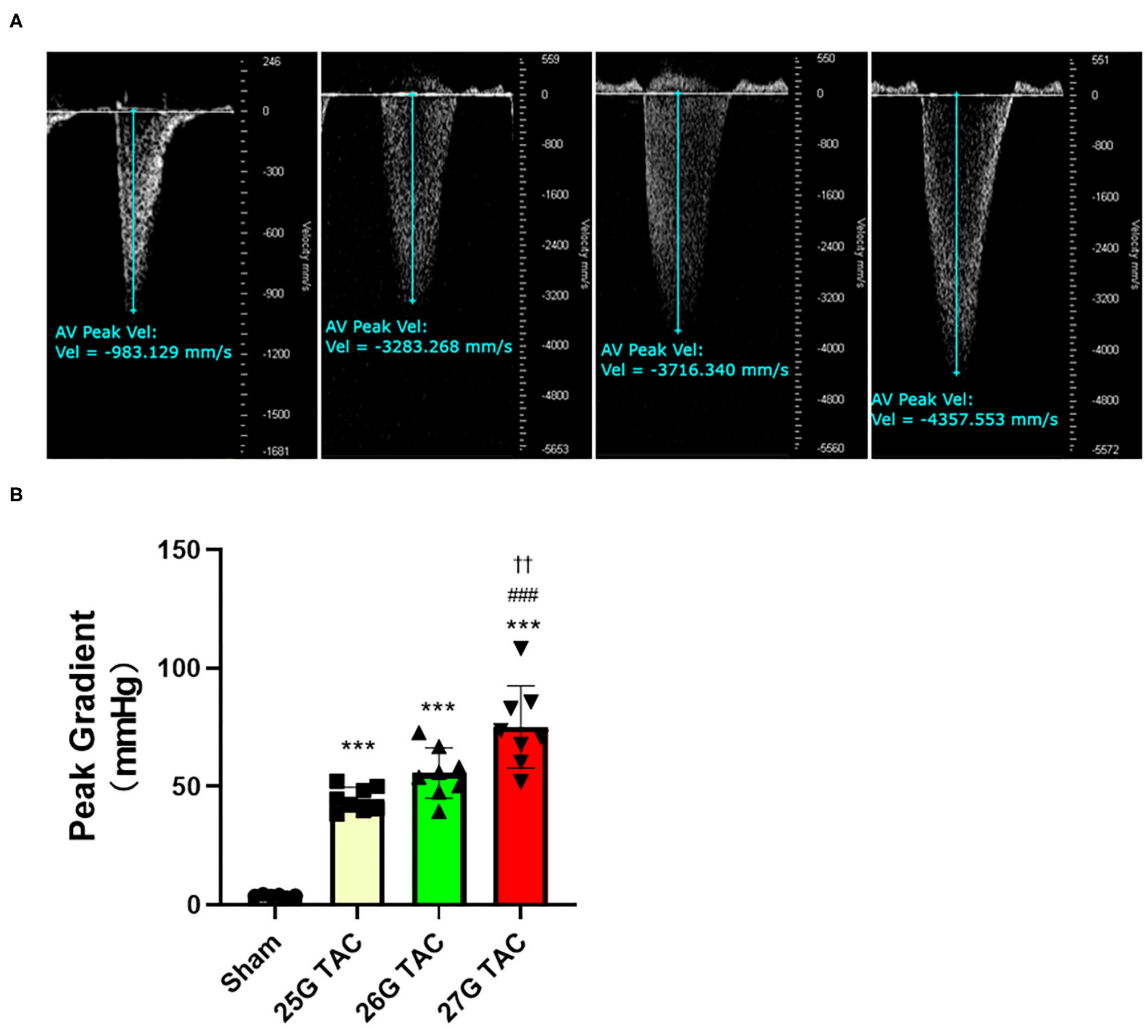
Aortic pressure gradients following 1 week of TAC. **(A)** Representative pulsed wave Doppler images for sham, 25, 26, and 27 G TAC mice after 1 week. **(B)** Peak pressure gradients calculated from Doppler velocities after 1 week of TAC. ***P < 0.001 vs. sham, ^###^*P* < 0.001 vs. 25 G TAC, ^††^*P* < 0.01 vs. 26 G TAC by one-way ANOVA with Tukey's post-test.

To evaluate the outcomes of different needle sizes on TAC-induced pressure overload conditions, we measured the pressure gradient across the construction site in each group using pulse wave Doppler. The pressure gradient was measured 1 week after TAC surgery. Compared with sham mice (3.8 ± 0.4 mmHg), the pressure gradient was significantly increased by TAC surgery in 25 G TAC (44.5 ± 1.8 mmHg), 26 G TAC (55.7 ± 3.7 mmHg), and 27 G TAC (75.2 ± 6.1 mmHg) (*p* < 0.001; Figures 1A,B). Moreover, the pressure gradient was significantly higher in 27 G TAC compared with 25 G TAC (*p* < 0.001) and 26 G TAC (*p* < 0.01). Although there was no statistically significant difference between 25 and 26 G TAC, the pressure gradient in 26 G TAC was much higher than that in 25 G TAC. These results show that the different needle sizes have successfully induced different degrees of pressure overload conditions in C57BL/6N mice.

### Cardiac Hypertrophy

**Figure 2:**
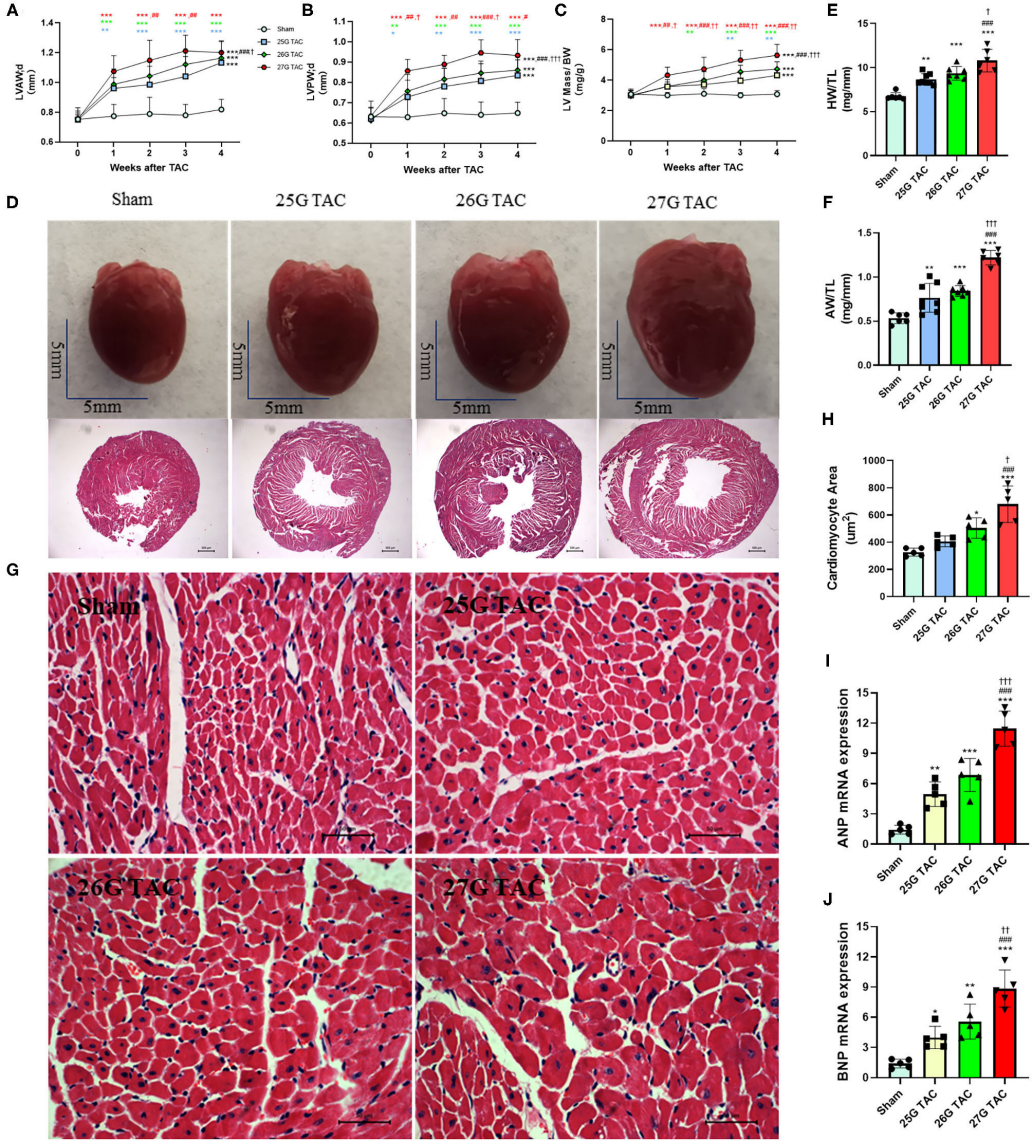
25, 26, and 27 G TAC induced different degrees of cardiac hypertrophy in C57BL/6N mice. The echocardiography-derived results of **(A)** the LVAW thickness, **(B)** LVPW thickness, and **(C)** LV mass normalized to body weight. **(D)** Representative heart pictures for sham, 25, 26, and 27 G TAC mice after 4 weeks of TAC and representative images of H&E staining of cross-heart sections; scale bars 500 um. **(E)** HW/TL and **(F)** AW/TL in sham, 25, 26, and 27 G TAC mice after 4 weeks of TAC. **(G)** Representative LV sections stained with H&E and DAPI (blue) for assessment of cardiomyocyte area. Scale bar= 50 μm. **(H)** Quantification of cardiomyocyte area. **(I,J)** RT-PCR analysis of ANP and BNP mRNA. *P < 0.05, **P < 0.01, and ***P < 0.001 vs. sham, ^#^*P* < 0.05, ^##^*p* < 0.01, ^###^*p* < 0.001 vs. 25 G, ^†^*P* < 0.05, ^††^*P* < 0.01, and ^†††^*P* < 0.001 vs. 26 G TAC, TAC by two- **(A–C)** or one-way ANOVA **(E,F,H)** with Tukey's post-test. Colored symbols **(A–C)** indicate *P < 0.05, **P < 0.01, and ***P < 0.001 vs. sham, ^#^*P* < 0.05, ^##^*p* < 0.01, ^###^*p* < 0.001 vs. 25 G, ^†^*P* < 0.05, ^††^*P* < 0.01 vs. 26 G TAC at the time point indicated.

Compared with sham mice, all mice subjected to TAC surgery successfully developed cardiac hypertrophy (Figure 2). The echocardiography results showed that the left ventricular anterior wall (LVAW) thickness in 25, 26, and 27 G TAC increased 25.2, 29.2, and 36.8%, respectively, compared with sham mice after 4 weeks of TAC (Figure 2A). Importantly, the LVAW thickness in 27 G TAC was much thicker than that in 26 (*p* < 0.05) and 25 G TAC (*p* < 0.001). Although there was no statistically significant difference in LVAW wall thickness between 25 and 26 G TAC, the LVAW thickness in 26 G TAC was increased by 3.8% compared with 25G TAC. Further *post-hoc* analysis showed a significant increase of LVAW thickness between all three TAC and sham mice after just 1 week of TAC surgery; however, there was no significant difference among the three TAC groups, whereas the significant difference between 25 and 27 G TAC emerged 2 and 3 weeks after TAC surgery, but this significant difference disappeared at 4 weeks after TAC surgery. Similar results about the left ventricular posterior wall (LVPW) thickness (Figure 2B) have been observed. LV mass normalized to body weight is one of the very important parameters of cardiac hypertrophy. Our results showed that LV mass increased 21.7, 29.3, and 49.2% in 25, 26, and 27 G TAC, respectively, compared with sham mice after 4 weeks of TAC surgery (Figure 2C). The mice subjected to 27 G TAC had more significant LV mass than 26 G TAC (*p* < 0.001), and 26 G TAC had more than that in 25 G TAC but with no statistical significance. Further *post-hoc* analysis showed that LV mass of the 27 G TAC mice significantly increased just 1 week after TAC surgery compared with sham mice; however, this significant difference appeared 2 weeks after TAC surgery in 26 G TAC and emerged 3 weeks after TAC in 25 G TAC mice.

In accordance with the echocardiographic data, the heart size was increased in 25, 26, and 27 G TAC compared with sham mice, and the heart size in 27 G TAC was bigger than that in 26 G TAC and the 26 G TAC bigger than that in 25 G TAC (Figure 2D). Similarly, the heart weight normalized to tibia length (HW/TL) and atrial weight normalized to tibia length (AW/TL) were all significantly increased in 25 (*p* < 0.01), 26 (*p* < 0.001), and 27 G TAC mice (*p* < 0.001) compared with sham mice (Figures 2E,F). Among the TAC mice, the HW/TL in 27 G TAC was significantly higher than that in 26 (*p* < 0.05) and 25 G TAC (*p* < 0.001); however, there was no significant difference between 25 and 26 G TAC. Accordingly, the cardiomyocyte area was significantly increased in 27 (*P* < 0.001) and 26 G TAC (*P* < 0.05) compared with sham mice, but the significant difference was absent in 25 G mice compared with sham mice (Figures 2G,H). What's more, the cardiomyocyte area in 27 G TAC mice was significantly larger than that in 25 (*p* < 0.001) and 26 G TAC mice (*p* < 0.05).

We also examined the expression of hypertrophic genes. Our results show that atrial natriuretic peptide (ANP) and B-type natriuretic peptides (BNP) gene expression was significantly increased in 25, 26, and 27 G TAC mice compared with sham mice, and the ANP and BNP gene expression in 27 G TAC was significantly higher than that in 26 and 25 G TAC; however, there was no significant difference between 25 and 26 G TAC (Figures 2I,J).

Taken together, these results show that 25, 26, and 27 G TAC successfully induced different degrees of cardiac hypertrophy.

### Cardiac Function

**Figure 3:**
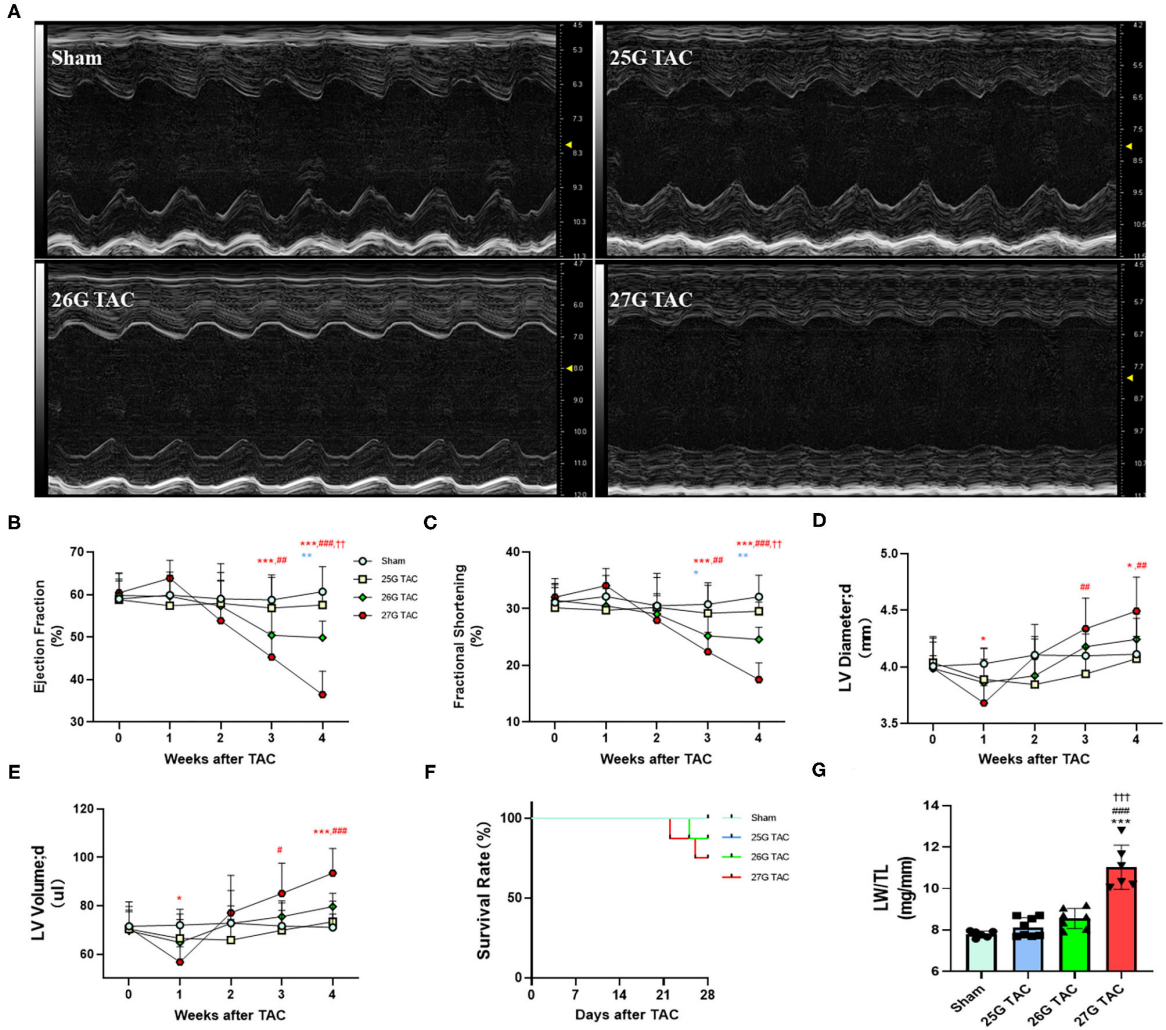
25, 26, and 27 G TAC induced different degrees of reduction of cardiac function in C57BL/6N mice. The echocardiography-derived results of **(A)** representative images of M-Mode images after 4 weeks of TAC show systolic dysfunction, **(B)** EF, **(C)** FS, **(D)** the end-diastolic LV diameter, and **(E)** the end-diastolic LV volume. **(F)** Kaplan–Meier survival curves. **(G)** LW/TL. ^***^P < 0.001 vs. sham, ^###^*p* < 0.001 vs. 25 G, ^†††^*P* < 0.001 vs. 26 G TAC by two- **(B–E)** or one-way ANOVA **(G)** with Tukey's post-test. Colored symbols **(A–C)** indicate ^*^P < 0.05, ^**^P < 0.01, and ^***^P < 0.001 vs. sham, ^#^*P* < 0.05, ^##^*p* < 0.01, ^###^*p* < 0.001 vs. 25 G, ^††^*P* < 0.01 vs. 26 G TAC at the time point indicated.

To test the cardiac function change induced by different degrees of constriction in the TAC surgery, LV structure and systolic function were analyzed presurgery and at 1, 2, 3, and 4 weeks after TAC surgery by using echocardiography. M-Mode analysis in the parasternal long axis detected a progressive decline in systolic function for mice subjected to 27 and 26 G TAC surgery from 2 to 4 weeks after TAC. There was a mild increasing trend at 1 week after TAC; however, there was no significant change in systolic function for mice subjected to 25 G TAC compared with sham mice (Figures 3A–C). The ejection fraction (EF) in 27 G TAC mice was significantly decreased at 3 weeks after TAC compared with sham mice, whereas this significant difference emerged in 26 G TAC mice at 4 weeks after TAC (Figure 3B). Importantly, the EF in 27 G TAC mice was much lower than that in 26 G TAC mice (*p* < 0.01) and in 25 G TAC mice (*p* < 0.001). Similar results were observed for the fractional shortening (FS) (Figure 3C). We also detected the end-diastolic LV diameter and volume, which are important parameters for LV dilation and cardiac dysfunction.

The end-diastolic LV diameter in mice subjected 27 G TAC was significantly decreased at 1 week after TAC surgery compared with sham mice and then progressively increased from 2 to 4 weeks after TAC and emerged with a significant difference at 4 weeks (*p* < 0.05) after TAC compared with sham mice (Figure 3D). Although the end-diastolic LV diameter in mice subjected to 26 G TAC had an increasing trend from 2 to 4 weeks after TAC, there was no statistically significant difference compared with sham mice. However, compared with 25 G TAC mice, the end-diastolic LV diameter in 27 G TAC mice was significantly higher at 3 weeks (*p* < 0.01) and 4 weeks (*p* < 0.01) after TAC. We observed similar results for the end-diastolic LV volume (Figure 3E). We also did the survival analysis after TAC surgery (Figure 3F). The mice subjected to 25 G TAC had 100% survival within 4 weeks after TAC, whereas one of eight mice (12.5%) subjected to 26 G TAC and two of eight mice (25%) subjected to 27 G TAC died 3–4 weeks after TAC. Moreover, we also analyzed the LW/TL to evaluate HF at 4 weeks after TAC (Figure 3G). The LW/TL was significantly increased in the mice subjected to 27 G TAC compared with sham mice (*p* < 0.001), 25 G TAC mice (*p* < 0.001), and 26 G TAC mice (*p* < 0.001). Although there was no statistically significant difference in LW/TL for 25 and 26 G TAC compared with sham, the LW/TL slightly increased 4.3 and 9.7% in 25 and 26 G TAC mice compared with sham mice, respectively.

### Cardiac Fibrosis

**Figure 4:**
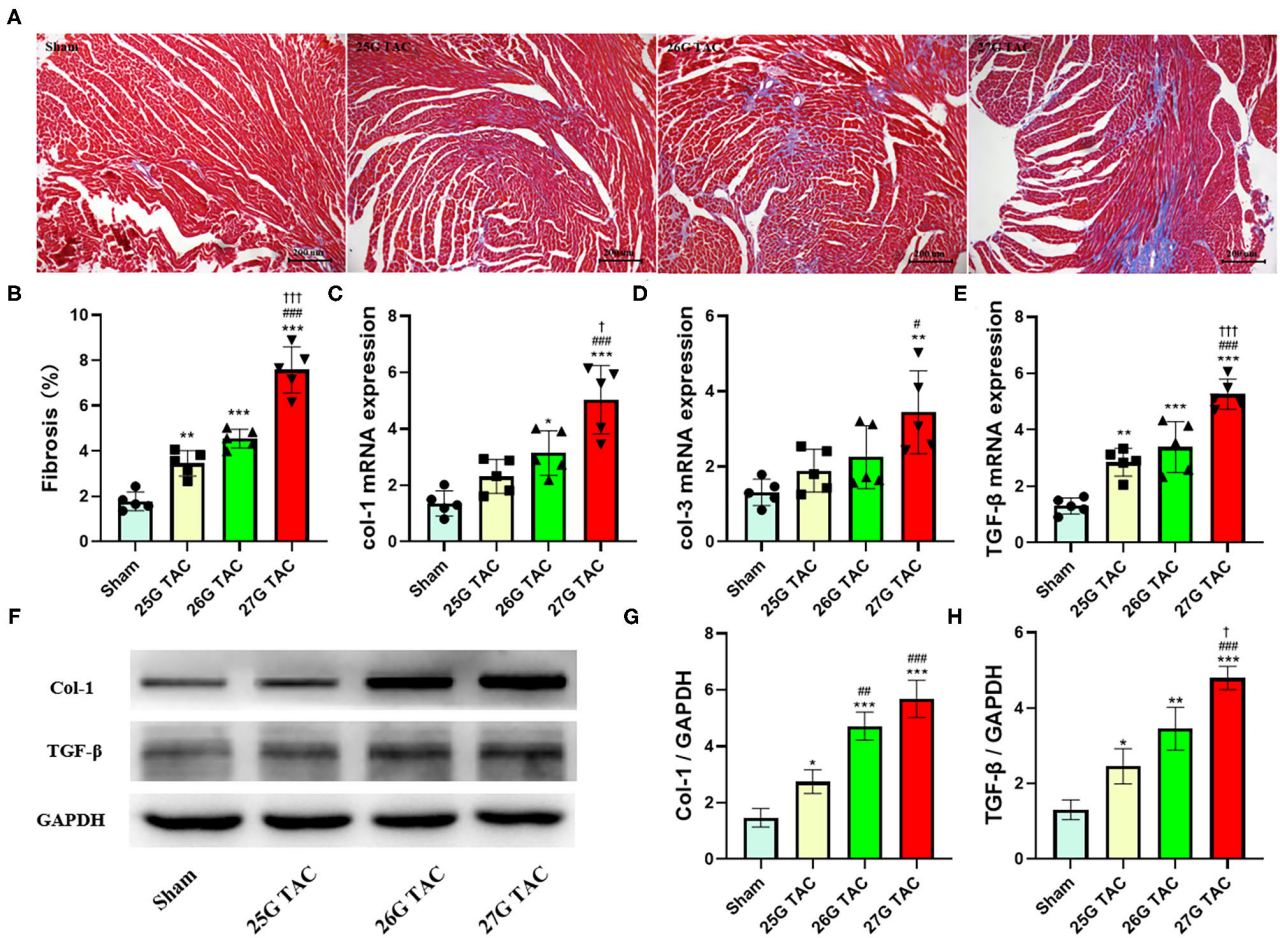
25, 26, and 27 G TAC induced different degrees of cardiac fibrosis in C57BL/6N mice. **(A)** Representative images for cardiac fibrosis. **(B)** Quantification of cardiac fibrosis. Apical left ventricular gene expression of **(C)** col-1, **(D)** col-3, **(E)** TGF-β. **(F–H)** Western blot analysis of col-1 and TGF-β. *P < 0.05, **P < 0.01, and ***P < 0.001 vs. sham, ^#^*P* < 0.05, ^##^*p* < 0.01, ^###^*p* < 0.001 vs. 25 G, ^†^*P* < 0.05, ^†††^*P* < 0.001 vs. 26 G TAC by one-way ANOVA with Tukey's post-test.

To test the cardiac fibrosis induced by different degrees of constriction in TAC surgery in mice, Masson's trichrome staining was applied to heart tissue paraffin sections. Compared with sham mice, mice subjected to 25 (*p* < 0.01), 26 (*p* < 0.001), and 27 G TAC (*p* < 0.001) developed significant cardiac fibrosis (Figures 4A,B). Importantly, mice subjected to 27 G TAC had much severer cardiac fibrosis than in 25 (*p* < 0.001) and 26 G TAC mice (*p* < 0.001). Accordingly, we observed similar results from the analysis of heart tissue fibrosis gene expression. The col-1, col-3, and TGF-β gene expression in 27 G TAC mice was significantly increased compared with sham mice, and the col-1 and TGF-β gene expression in 26 G mice were significantly increased compared with sham mice (Figures 4C–E). The col-1 and TGF-β gene expression in 27 G TAC mice was significantly higher than that in 25 and 26 G TAC mice. We got similar results from analysis of col-1 and TGF-β protein expression (Figures 4F–H); the protein expression of col-1 and TGF-β in 27 G TAC mice was significantly higher than in 25 and 26 G TAC mice. Although not all data showed a significantly difference, the fibrosis gene expression showed a gradually increasing trend in the order of sham mice, 25 G TAC mice, 26 G TAC mice, and 27 G TAC mice.

## Discussion

In the present study, we directly compared the different cardiac remodeling phenotypes induced by different degrees of LV pressure overload in C57BL/6N mice. Specifically, we analyzed the different phenotypes induced by 25, 26, and 27 G TAC in c57BL/6N mice in terms of pressure gradient, cardiac hypertrophy, cardiac function, HF situation, survival condition, and cardiac fibrosis. All C57BL/6N mice subjected to TAC surgery developed significant hypertrophy. Mice subjected to 27 G TAC experienced a compensatory period to decompensatory period over 4 weeks after TAC, and 27 G TAC mice had severe cardiac dysfunction, severe cardiac fibrosis, and exhibited characteristics of heart failure at 4 weeks post-TAC. Whereas, mice subjected to 26 G TAC showed a much milder response in cardiac dysfunction, cardiac fibrosis compared to 27 G TAC, and a very small fraction of 26 G TAC mice exhibited characteristics of HF, which means 26 G TAC caused the transition from the LV hypertrophy to an early phase of cardiac dysfunction and HF in C57BL/6N mice. However, except for significant cardiac hypertrophy, there was no obvious cardiac dysfunction, cardiac fibrosis, and characteristics of HF observed in 25 G TAC mice. Based on our results, we conclude that the 25, 26, and 27 G TAC induced distinct phenotypes in C57BL/6N mice.

TAC surgery is one of the most popular pressure overload models for studying cardiac hypertrophy and cardiac remodeling, and it has been widely used and modified to be less invasive since it was first reported in 1991 (4, 6–9, 17, 18). There are many factors that affect the phenotype of TAC-induced cardiac remodeling, among which the constriction degree is the most important one. Besides this, species of animals, age, sex, and genetic background also affect the phenotype variability of TAC-induced cardiac remodeling. The widely phenotypic difference reported with this model has raised the question of how we can choose the proper condition of TAC surgery in a specific study. Unfortunately, very few studies focus on the direct comparison of different cardiac remodeling phenotypes induced by 25, 26, and 27 G TAC, and no one has compared this difference in C57BL/6N mice. C57BL/6N and C57BL/6J mice are two of the most commonly used C57BL/6 substrains, and they have been widely used as genetic background transgenic and gene knockout mice. Michelle and colleagues conducted a comparative phenotypic and genomic analysis of C57BL/6J and C57BL/6N mouse strains, and annotated 34 SNPs and two indels that distinguish C57BL/6J and C57BL/6N coding sequences (19). Besides this, it is reported that there are phenotypic differences between C57BL/6J and C57BL/6N in many disease models, such as the diet-induced type 2 diabetes model (20), the non-alcoholic steatohepatitis model (21), influenza a virus induced inflammation-associated disease (22), and postnatal hypoxic-ischemic brain injury mouse model (23) as well as pressure overload–induced cardiac hypertrophy (24). Lorena and colleagues found that cardiac response to pressure overload is distinct between C57BL/6J and C57BL/6N mice, and the survival and cardiac function are significantly lower in C57BL/6N mice compared with C57BL/6J (24). However, to our knowledge, a direct comparison of pressure gradient, cardiac hypertrophy, cardiac function, and cardiac fibrosis phenotype changes among the 25, 26, and 27 TAC-induced cardiac remodeling have not been reported.

Cardiac hypertrophy is the consequence of the heart's response to a variety of extrinsic and intrinsic stimuli, which impose increased biomechanical stress (25). Initially, the development of hypertrophy is a compensatory mechanism, and contractile function is maintained. However, when the heart is subjected to excessive and/or persistent stress, cardiac function becomes maladaptive and decompensatory, eventually leading to HF (26). Constriction degree is an important factor that affects the development process of pressure overload–induced cardiac remodeling. In the present study, we directly compared the phenotypes of cardiac remodeling induced by 25, 26, and 27 G TAC in C57BL/6N mice. We found that mice subjected to 27 G TAC surgery had a short increasing trend of cardiac function at 1 week after TAC, then the cardiac function continuously decreased from 2 weeks after TAC surgery, and eventually developed HF characterized by significantly decreased cardiac function, significantly increased lung mass and atrial weight, and more mortality at 4 weeks after TAC. This data indicated that mice subjected to 27 G TAC surgery experienced both compensatory-phase and decompensatory-phase cardiac hypertrophy, which suggests 27 G TAC surgery in C57BL/6N mice is extremely useful in both mechanistic and drug studies aiming to improve or reverse the cardiac dysfunction in a pressure overload–induced heart failure model. Compared with 27 G TAC mice, mice subjected to 26 G TAC surgery had less cardiac dysfunction and only a very small fraction of mice showed signs of HF at 4 weeks after TAC surgery, which indicated that 26 G TAC mice were at the transition stage between compensated and decompensated heart failure. In this situation, adverse cardiac remodeling was still observed, but the mortality can be maintained at a low level, which probably makes 26 G TAC surgery more useful on the mice with more susceptibility to mortality, such as genetically modified mice. In contrast, mice subjected to 25 G TAC surgery were still at the compensatory cardiac hypertrophy stage without cardiac dysfunction and HF. Therefore, the 25 G TAC model could be very useful for studying hypertensive heart disease in mice.

Cardiac fibrosis is defined as the deposition of extracellular matrix proteins in the cardiac interstitium, and interstitial fibrosis plays an important role in the development and progression of HF via causing adverse electrical and mechanical disturbances in diseased hearts (27). Pressure overload–induced cardiac hypertrophy is invariably accompanied by the formation of cardiac fibrosis (28), and this cardiac fibrosis alters myocardial stiffness and consequently affects myocardial function, which plays a major role in the progressive decompensation of the cardiac dysfunction (29, 30). Here, we also analyzed the cardiac fibrosis difference in C57BL/6N mice subjected to 25, 26, and 27 G TAC surgery. Mice subjected to 27 G TAC had severe cardiac fibrosis confirmed by the analysis of Masson's trichrome staining and fibrosis-related gene expression. Compared with 27 G TAC mice, the cardiac fibrosis in 26 G TAC mice was much milder. Although there was slight cardiac fibrosis in 25 G TAC mice, there was not much difference compared with sham mice. The cardiac fibrosis is consistent with the cardiac function changes in 25, 26, and 27 G TAC mice, respectively, which confirms the importance of cardiac fibrosis in the development and progression of HF.

The present study provides a comprehensive analysis of distinct phenotypes induced by 25, 26, and 27 G TAC surgery in C57BL/6N mice. However, there are still some limitations in the current study. First, all mice used in the present study were male C57BL/6N mice aged 10 weeks although the cardiac remodeling phenotypes induced by TAC surgery are affected by the sex and age of animals; therefore, the results in the present study may not apply to female or different age mice. Given the consideration of most previously published papers, we chose to focus our study within 4 weeks after TAC surgery although we got distinct phenotypes from 25, 26, and 27 G TAC mice at this time point in terms of cardiac hypertrophy, cardiac function, and cardiac fibrosis; however, we did not analyze the phenotype changes among the different degree TAC over 4 weeks after TAC, so whether these distinct phenotypes among the three different degrees of TAC group are magnified or blunted more than 4 weeks after TAC is still unknown. In addition, the mice subjected to 25 G TAC surgery in current study were still at the compensatory cardiac hypertrophy stage, so we have no idea about the specific timeline for the development of pressure overload–induced heart failure in 25 G TAC mice, and this question should be addressed in the future study.

In summary, in the present study, we directly compared the different phenotypes induced by 25, 26, and 27 G TAC surgery in the aspects of pressure gradient, cardiac hypertrophy, cardiac function, and HF and cardiac fibrosis. Importantly, this is the first time to conduct such a study in C57BL/6N mice, and our results will provide important reference value especially for researchers who conduct pressure overload–induced cardiac hypertrophy studies using C57BL/6N mice or genetically modified mice with a C57BL/6N background.

## Data Availability Statement

The original contributions presented in the study are included in the article/Supplementary Material, further inquiries can be directed to the corresponding author/s.

## Ethics Statement

The animal study was reviewed and approved by Animal Care and Use Committee of Fudan University.

## Author Contributions

HD and L-LM performed the experiments and analyzed the data. HD wrote the manuscript. F-JK and ZQ designed and supervised the study and revised the manuscript. All authors contributed to the article and approved the submitted version.

## Conflict of Interest

The authors declare that the research was conducted in the absence of any commercial or financial relationships that could be construed as a potential conflict of interest.

## Supplementary Material

Supplementary Figure 1 Western blot analysis of p-Erk. **(A)** Representative images for p-Erk, t-Erk, and GAPDH. **(B)** Quantification of p-Erk/t-Erk. ^*^*P* < 0.05, ^***^*P* < 0.001 vs. sham, ^##^*P* < 0.01, ^###^*p* < 0.001 vs. 25 G, ^††^*P* < 0.01 vs. 26 G TAC by one-way ANOVA with Tukey's post-test.
