# Supplementary figures and images for: Distinct Phenotypes Induced by Different Degrees of Transverse Aortic Constriction in C57BL/6N Mice
Source: Front Cardiovasc Med. 2021 Apr 22;8:641272. doi: 10.3389/fcvm.2021.641272 (PMC8100039; doi:10.3389/fcvm.2021.641272)

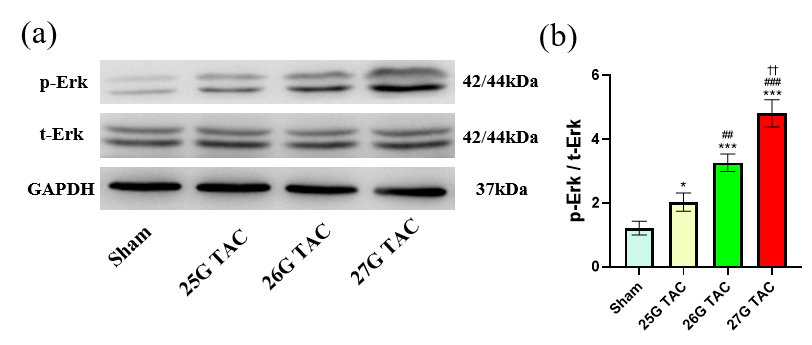

Supplement: Supplementary Figure 1 — Western blot analysis of p-Erk. (A) Representative images for p-Erk, t-Erk, and GAPDH. (B) Quantification of p-Erk/t-Erk. *P < 0.05, ***P < 0.001 vs. sham, ##P < 0.01, ###p < 0.001 vs. 25 G, ††P < 0.01 vs. 26 G TAC by one-way ANOVA with Tukey's post-test. [file Image_1.TIF]
